# Microlobulated Margins, Internal Punctate High Echoes, and Enhancing Internal Septation As Important Diagnostic Clues for Invasive Solid Papillary Carcinomas of the Breast: A Case Report

**DOI:** 10.7759/cureus.110149

**Published:** 2026-06-02

**Authors:** Yuya Omura, Shoji Oura

**Affiliations:** 1 Department of Surgery, Kishiwada Tokushukai Hospital, Kishiwada, JPN

**Keywords:** breast cancer, enhancing internal septation, internal punctate high echoes, invasive solid papillary carcinoma, microloburated margins

## Abstract

Invasive solid papillary carcinoma (ISPC) of the breast is a rare disorder and has few reported typical image characteristics to date. An 81-year-old woman was referred to our hospital for the detailed examination of a breast mass incidentally detected on computed tomography. Mammography showed a microlobulated mass, 1.6 cm in size. Ultrasound showed an oval mass with microlobulated margins, internal punctate high echoes, and slightly enhanced posterior echoes. Magnetic resonance imaging of the tumor showed low signals on T1-weighted images, weak high signals on fat-suppressed T2-weighted images, and early and retained enhancing internal septation on dynamic studies. After the pathological diagnosis of breast cancer, the patient underwent a partial mastectomy and a sentinel node biopsy. Postoperative pathological study showed that the tumor had cancer cells growing in a solid fashion around the fibrovascular stalks, stromal reaction in the central areas, and fat tissue invasion. Immunostaining of the tumor showed estrogen receptor, progesterone receptor, synaptophysin, CD56, and chromogranin positivity; human epidermal growth factor receptor type 2 and p63 negativities; and a Ki-67 labelling index of 5%, leading to the diagnosis of ISPC. Diagnostic physicians should note that ISPC may have microlobulated margins, internal punctate high echoes, slight posterior echo enhancement, slight high signals on fat-suppressed T2-weighted images, and enhancing internal septation.

## Introduction

Mammography can provide highly objective findings of breast disorders to diagnostic physicians. In short, spiculated mass and fine linear calcifications can easily and directly lead to the evaluation of Breast Imaging Reporting and Data System category 5 [1]. Mammography, therefore, has long played the mainstay in the diagnosis of breast cancer [2].

Ultrasound cannot show the whole breast, but can provide clear cross-sectional images of breast masses even in the dense breast, which often makes the depiction of breast disorders difficult on mammography. Ultrasound can sometimes detect the ductal spread of cancer cells, which mammography cannot depict except for the cases having microcalcifications in a linear or segmental fashion [3].

Magnetic resonance imaging (MRI) is another important diagnostic test for breast disorders. The washout pattern on dynamic studies highly indicates the malignant cell components in the target masses. Conversely, plateau and persistent patterns suggest benign disorders [4]. In addition, MRI can depict ductal spread and daughter nodules more clearly than mammography and ultrasound.

Invasive solid papillary carcinoma (ISPC) of the breast is a rare disorder and has been evaluated for its clinico-pathological characteristics in some studies, suggesting that ISPCs generally have estrogen receptor (ER) positivity and are often detected in small sizes [5,6]. One case report described the internal echoes of the ISPC as hypoechoic, even though it was actually hyperechoic [7]. Another case report also described the internal echoes of the ISPC as hypoechoic, but in reality, punctate high echoes were present [8]. Few studies, therefore, have evaluated the characteristic image findings of ISPC based on the pathological findings to date.

We herein report a rare ISPC of the breast and its characteristic image findings closely correlated with the pathological characteristics of it.

## Case presentation

An 81-year-old woman was referred to our hospital for the detailed examination of the right breast mass, which was incidentally detected on computed tomography for suspected lung cancer. The patient had no family history of breast or ovarian cancer, nor any history of major disorders, including malignant tumors. We palpated a small mass in the inner and upper quadrant of the right breast. Mammography showed a mass of 1.6 cm in size with microlobulated margins (Figure 1).

**Figure 1:**
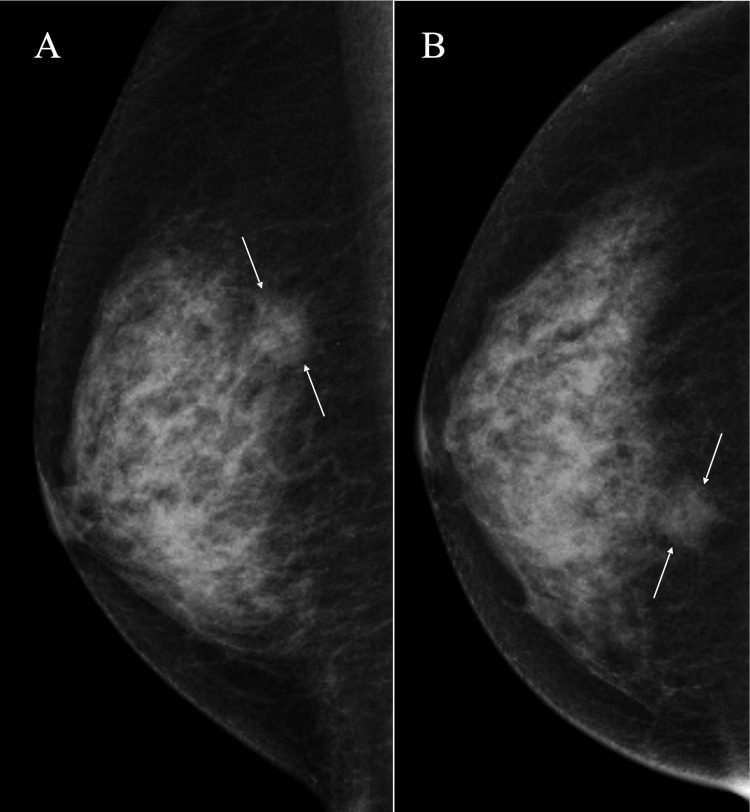
Mammography findings A. Medio-lateral oblique view mammography showing a mass (arrows) with microlobulated margins in the upper part of the breast. B. Cranio-caudal view mammography showing the mass (arrows) in the inner part of the breast.

Ultrasound showed an oval mass with microlobulated margins, internal punctate high echoes, and slightly enhanced posterior echoes (Figure 2).

**Figure 2:**
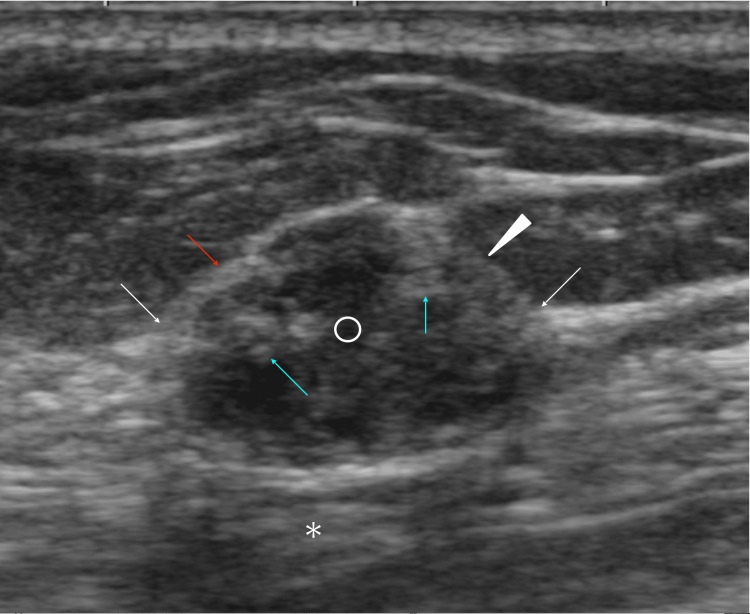
Ultrasound findings Ultrasound showing that an oval mass (open circle) protruded forward beyond the anterior borders of the mammary gland (white arrows) and had internal punctate high echoes (blue arrows), microlobulated margins (arrowhead), presumed haloes around the mass (red arrow), and slightly enhanced posterior echoes (asterisk).

MRI of the tumor showed low signals on T1-weighted images, weak high signals on fat-suppressed T2-weighted images, and early and retained enhancing internal septation on dynamic studies (Figure 3).

**Figure 3:**
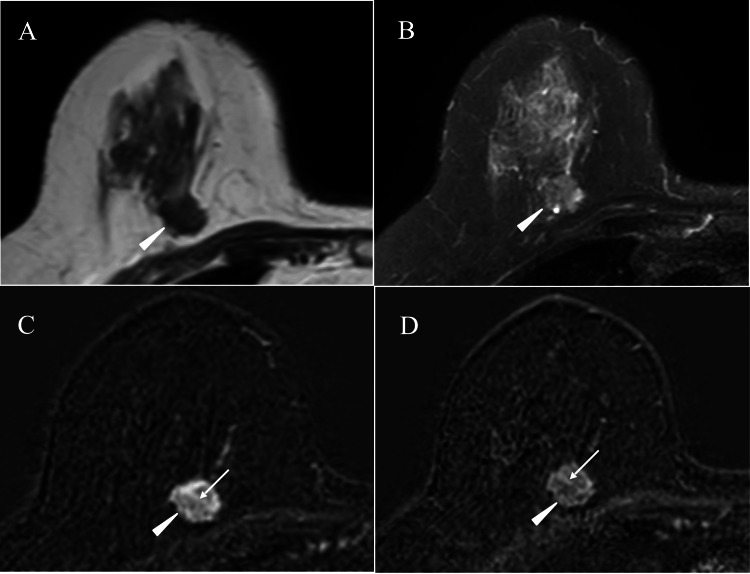
Magnetic resonance imaging (MRI) findings MRI of the tumor (arrowhead) showing low signals on T1-weighted images (A), weak high signals on fat-suppressed T2-weighted images (B), and early (C) and retained (D) enhancing internal septation (arrow) on dynamic studies.

We, therefore, did a core needle biopsy of the breast mass under the tentative diagnosis of breast cancer. Core needle biopsy pathologically showed solid cancer cells growing in an expansive fashion, accompanied by fibrovascular stalks, leading to the diagnosis of ISPC. Immunostaining of the ISPC showed both ER and progesterone receptor (PgR) positivity (Allred score 8), human epidermal growth factor receptor type 2 negativity, synaptophysin, CD56, and chromogranin positivity, and a Ki-67 labelling index of 5%. Absence of both daughter nodules and extensive ductal spread on images made her undergo a partial mastectomy and sentinel node biopsy. Frozen and permanent sections showed that the tumor had node and margin negativities, consisted of cancer cells growing in a solid fashion around the fibrovascular stalks, had stromal reaction within the mass, and had cancer cell infiltration into the surrounding fat tissue (10 mm in invasive size, nuclear grade 1, stage 1). Immunostaining showed similar results to those of the core needle biopsy specimen about ER, PgR, HER2, and Ki-67 labelling index, and p63 negativity (Figure 4).

**Figure 4:**
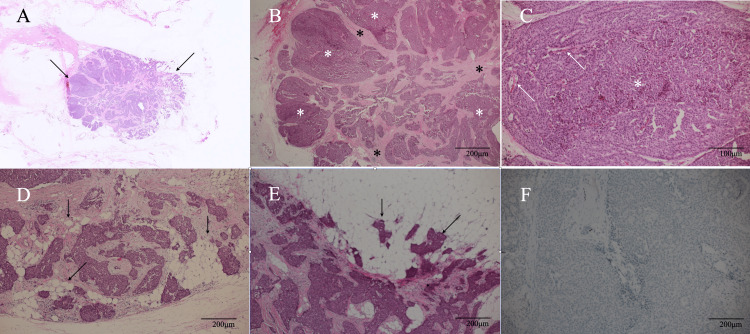
Pathological findings A. Low-magnified view showing that solid cancer foci aggregated and formed the tumor (arrows) (HE ×4). B. Magnified view showing that solid cancer foci (white asterisks) aggregated closely with the intervening fibrous tissue (black asterisks) (HE ×100). C. Magnified view showing that cancer cells were growing in a solid fashion (asterisk) around the fibrovascular stalks (arrows) (HE ×200). D. Magnified view showing that the tumor had many fat cells (arrows) within it (HE ×100). E. Magnified view showing that cancer cells (arrows) spread into the peritumoral fat tissue (HE ×100). F. Immunostaining clarifying no positive cells for p63 (×100).

The patient recovered uneventfully, was discharged on the second day after surgery, started receiving an aromatase inhibitor, has been well for two months, and is scheduled for 10-year follow-ups on an outpatient basis.

## Discussion

The present ISPC had microlobulated margins both on mammography and ultrasound. It is well known that ISPCs have many solid cancer foci with stromal reaction, which show a so-called geographical jigsaw pattern [5]. ISPCs, therefore, generally have microlobulated margins, which are formed by multiple solid cancer nests growing in an expansive fashion and fibrous connective tissue filling the space among cancer nests.

Breast cancer, such as solid type invasive ductal carcinoma and medullary carcinoma, has a hypoechoic or severely hypoechoic pattern due to little ultrasound wave backscattering caused by the similarity of acoustic impedance among cancer cells with similar morphology [9]. ISPCs have both solid cancer structures and papillary structures. The latter contains some kind of microvoids in their basic structures, which cause ultrasound wave backscattering and can generate internal punctate high echoes [10]. Fat cells originally located in the breast presumably remain in the interspaces among solid cancer foci, showing the geographical jigsaw pattern in this case. Fat tissue, known as the pathological component having the least acoustic impedance in the body, also generates a strong ultrasound wave backscattering when facing cancer cells and other pathological components, also leading to the internal punctate high echo formation in the mass [11,12]. Internal punctate high echoes, therefore, can be an important ultrasound finding of ISPCs.

The present ISPC had early and retained enhancing internal septation [12]. It is well known that breast cancer with fibrous components has a plateau or persistent enhancement pattern on dynamic studies. Enhancing internal septation well matched to the fibrous component areas in this ISPC. Enhancing internal septation, therefore, may be another important imaging finding in ISPC of the breast.

The present ISPC had slightly enhanced posterior echoes [13]. Cancer cell-rich tumors generally have enhanced posterior echoes due to less attenuation of ultrasound waves. A small amount of fibrous components generally observed in ISPCs can somewhat attenuate ultrasound waves and, therefore, lessen posterior echo enhancement. In addition, fat cell interminglement made the tumor more hypo-intense on fat-suppressed T2-weighted images.

ISPCs generally have ER positivity and indolent characteristics, making them often detected in small sizes, like this case [5]. Ultrasound findings mentioned above resemble those of intramammary lymph nodes. In fact, we initially included the intramammary lymph node as a possible diagnosis for this tumor. MRI evaluation, however, easily led us to the diagnosis of suspected breast cancer. Diagnostic physicians, therefore, should add MRI evaluation when diagnosing small breast masses with possible intramammary lymph nodes for the avoidance of under evaluation.

## Conclusions

This study examined the correlation between image and pathological findings based only on one ISPC case. Pathological evaluation of it, however, suggests that ISPCs may have microlobulated margins, internal punctate high echoes, slightly enhanced posterior echoes, weak high signals on fat-suppressed T2-weighted images, and enhancing internal septation. Diagnostic physicians should further note that ultrasound findings of ISPC often resemble those of intramammary lymph nodes. Diagnostic physicians, therefore, should evaluate intramammary lymph node-like breast masses with MRI to avoid misdiagnosis. Further research involving a larger number of ISPC cases is warranted.
